# Phytoprofiling of *Sorbus* L. Inflorescences: A Valuable and Promising Resource for Phenolics

**DOI:** 10.3390/plants11243421

**Published:** 2022-12-07

**Authors:** Kristina Zymone, Lina Raudone, Vaidotas Žvikas, Valdas Jakštas, Valdimaras Janulis

**Affiliations:** 1 Department of Analytical and Toxicological Chemistry, Lithuanian University of Health Sciences, Sukileliu Av. 13, LT-50162 Kaunas, Lithuania; 2 Laboratory of Biopharmaceutical Research, Institute of Pharmaceutical Technologies, Lithuanian University of Health Sciences, Sukileliu Av. 13, LT-50162 Kaunas, Lithuania; 3 Department of Pharmacognosy, Lithuanian University of Health Sciences, Sukileliu Av. 13, LT-50162 Kaunas, Lithuania

**Keywords:** *Sorbus* L., chlorogenic acid, phenolic compounds, sexangularetin, inflorescences, functional ingredients

## Abstract

The leaves and fruits of various *Sorbus* L. genotypes have long ethnopharmacological and food-usage histories, but inflorescences are still underutilized and neglected materials with scarce phytochemical scientific evidence. The aim of this study was to determine the phenolic profiles of inflorescence extracts of 26 *Sorbus* species, genotypes, and cultivars. HPLC and UPLS with MS detection were applied, and coupled data revealed unique phytochemical phenolic profiles. Neochlorogenic and chlorogenic acids were the key compounds, reaching up to 5.8 mg/g of dw. Rutin, isoquercitrin, quercetin 3*-O-*malonylglucoside, isorhamnetin 3*-O-*rutinoside, sexangularetin derivative, and kaempferol acetyl hexoside were detected in all *Sorbus* inflorescence samples. Overall, high quantitative heterogeneity across the various *Sorbus* genotypes was found by profiling. Phenolic fingerprint profiles and sexangularetin derivatives could serve as markers in authenticity studies and quality control schemes. The species *S. amurensis*, *S. arranensis*, *S. commixta*, and *S. discolor* and the cultivars ‘Chamsis Louing’, ‘Coral Beauty’, and ‘Edulis’ could be used as target genotypes for production of smart and innovative inflorescence matrix-based ingredients.

## 1. Introduction

Increasing consumers’ awareness about safe, health-promoting foods—and, specifically, the search for new safe and natural plant origin extracts and compounds that could be used with versatility in various industries for the production of cosmeceuticals, nutraceuticals, and pharmaceuticals—raises challenges and scientific uncertainties [1]. Furthermore, the increasing demand for innovative and functional food products with clear and clean labeling requires natural, safe, and simple ingredients that can be used conventionally [2]. Plant origin materials contain bodies of specialized metabolites that, when present in the extracts, exert health-promoting or pharmacological effects, as well as synergistic and additive effects [3]. In addition to the well-known plant materials with long ethnopharmacological histories, there are certain underutilized and neglected plants that can provide various raw materials that are extremely rich in particular groups of compounds of interest [4,5,6].

The genus *Sorbus* L. has a highly complicated systematics encompassing numerous subgenera and sections that determine its diversity sensu lato [7]. Furthermore, *Sorbus*’ extraordinary apomixis, cross-breeding, and intergeneric hybridization abilities have led to a great expansion of *Sorbus* genotypes, cultivars, hybrids, and varieties [8,9]. Almost all parts of the *Sorbus* plants have a historical record of usage in various traditional medicinal systems, as well as in food, fodder, beauty, health, and wellness-promoting products [10,11,12,13,14]. The fruits have been included in pharmacopoeias as polyvitaminic substances and in daily nutrition, but, due to their tart and specific taste, they are neglected or only used occasionally, although the ascorbic acid content exceeds the amount in oranges [4,15,16]. Nevertheless, their fruit powders could be incorporated as an ingredient in various processed foods and new products [4,17]. Extensive breeding and hybridization programs have led to several sweet-tasting rowanberries (*Sorbus* L. cultivars) that have potential as functional food ingredients containing amounts of carotenoids equivalent to commonly known sources, such as carrots [16,18,19,20]. Furthermore, due to their unique pattern of sugars and sugar alcohols, they could be incorporated in specialized food production [21,22]. Recent research has been devoted to elucidating the phytochemical richness and variability of phenolic profiles in the leaves of *Sorbus* species and varieties, as they contain more than fivefold higher amounts of phenolic compounds than commonly used fruits [23,24,25]. Although all *Sorbus* raw materials—namely, bark, leaves, and fruits—have been extensively investigated, the inflorescences are still one of the least studied raw materials. *Sorbus* plants contain decorative corymbs that cluster the dense flowers [26]. Scientific knowledge regarding the phytochemical composition of inflorescences is still limited. Olszewska et al. determined the phytochemical compositions of inflorescences of *S. aucuparia*, *S. commixta*, *S. decora*, *S. gracilis*, *S. koehneana*, *S. torminalis*, *S. pohuashanensis*, *S. sitchensis*, *S. intermedia*, and *S. aria* [23,24,25,27,28,29]. Recently, they produced a standardized inflorescence extract from *S. aucuparia* and determined its detailed phytochemical profile, as well as antioxidant and other biological properties [27]. However, the detailed profiling of inflorescences of other *Sorbus* species and cultivars has not yet been performed. Preliminary data suggest that *Sorbus* inflorescences contain significantly higher amounts of phenolic compounds than the fruits and even leaves [23,24,25,30]. Therefore, it is extremely important to determine their qualitative and quantitative profiles and define the analytical markers. Furthermore, the inflorescences of *Sorbus* contain the specific compound sexangularetin and its derivatives, which have not been detected in the fruits or leaves. These compounds could serve as authentication markers ensuring the quality of extracts [19]. 

Being climate- and environment-resilient, *Sorbus* plants are particularly attractive species for growth in standardized plantations, as they can withstand poor soils and harsh environments [31]. Sustainable and no-waste technologies could be applied for smart collection and preparation of *Sorbus* extracts from various raw materials rich in particular compounds [4,32].

The aim of this study was to determine the phenolic profiles of inflorescence extracts of 26 *Sorbus* L. species, genotypes, and cultivars. To the best of our knowledge, the profiles for the inflorescences of most species are reported for the first time. The research was focused on the phenolic profiles of the inflorescences, utilizing chemometric analysis to elucidate the chemophenetic differences and link the genotype with the pattern of phenolic acids and flavonoids. The lack of research on the phytochemical patterns of inflorescences of different *Sorbus* species highlights the need to elucidate the patterns and markers of the phenolic profiles. Phenolic compounds could act as chemical markers for the coupling or differentiation of species and substantiation of their parental origin. The peculiarities of the phenolic profiles could provide significant information for chemophenetic studies of this remarkably diverse genus of *Sorbus*. 

## 2. Results

### 2.1. Quantitative Phenolic Profiling of Sorbus L. Species and Cultivars

Analysis of the qualitative and quantitative composition of the phenolic compounds in the raw plant material samples of rowan inflorescences was performed. The chromatogram of ethanol extracts from the inflorescence samples of *Sorbus* plants is presented in Figure S1. The results of qualitative analysis of phenolic compounds are presented in Table S1. Neochlorogenic, chlorogenic, and cryptochlorogenic acids, as well as dicaffeoylquinic acid derivative 1 and dicaffeoylquinnic acid derivative 2, were identified in all inflorescence samples tested (Table 1). Caffeoylshikimic acid was not detected only in inflorescences of *S. aria* and *S. arranensis*, and coumaroylquinic acid derivative was detected only in certain inflorescence samples. The concentrations of these caffeoylquinic acids varied significantly among the tested samples, with differences of up to twentyfold. Chlorogenic acid was the predominant component in most of the inflorescence samples from the various species and cultivars, although the predominant component in the inflorescence samples of *S. arranensis* and *S. lancifolia* was neochlorogenic acid, while quercetin 3*-O-*malonylglucoside was the predominant component in the inflorescences of ‘Edulis’. The greatest amount of chlorogenic acid was found in the inflorescence samples of *S. commixta* (31,539 ± 2503 µg/g), *S. amurensis* (29,733 ± 1465 µg/g), and ‘Chamsis Louing’ (25,867 ± 1557 µg/g) (Table 1). The greatest concentrations of neochlorogenic acid (18,712 ± 1740 µg/g, 17,739 ± 2442 µg/g, and 17,011 ± 441 µg/g) were found in the inflorescence samples of ‘Coral Beauty’, ‘Carpet of Gold’, and *S. amurensis*, respectively (Table 1). 

Significant variation in the flavonol profiles depending on the *Sorbus* species/cultivar was determined (Table 2 and Table 3), with the amounts varying up to 33-fold. Rutin, isoquercitrin, quercetin 3*-O-*malonylglucoside, isorhamnetin 3*-O-*rutinoside, sexangularetin derivative, and kaempferol acetyl hexoside were detected in all *Sorbus* inflorescence samples. Isoquercitrin was the predominant component of the flavonoid complexes in the inflorescence samples of *S. commixta*, *S. x hostii*, ‘Alaja Krupnaja’, ‘Granatnaja’, ‘Koncentra’, ‘Krasnaja Nevezinskaja’, ‘Miciurinskaja Desertnaja’, ‘Nevezinskaja’, ‘Neveziskaja Zolotistaja’, ‘Nevezinskaja Zoltaja’, ‘Oranzevaja’, and ‘Titan’, while quercetin 3*-O-*malonylglucoside was the predominant component in the inflorescences of *S. aria*, *S. arranensis*, *S. lancifolia*, *S. hybrida* subsp. *Gotlandica*, *S. hybrida* subsp. *Persecta*, ‘Carpet of Gold’, ‘Chamsis Louing’, ‘Coral Beauty’, ‘Edulis’, ‘Pink Queen’, and ‘Yellow Upright’. We determined that, in the inflorescence samples of *S. discolor* and *S. semi-incisa*, the predominant flavonoid was rutin and, in the inflorescences of *S. amurensis*, it was hyperoside.

### 2.2. Hierarchical Cluster Analysis of Phenolic Compounds in Sorbus L. Species and Cultivars

**Figure 1:**
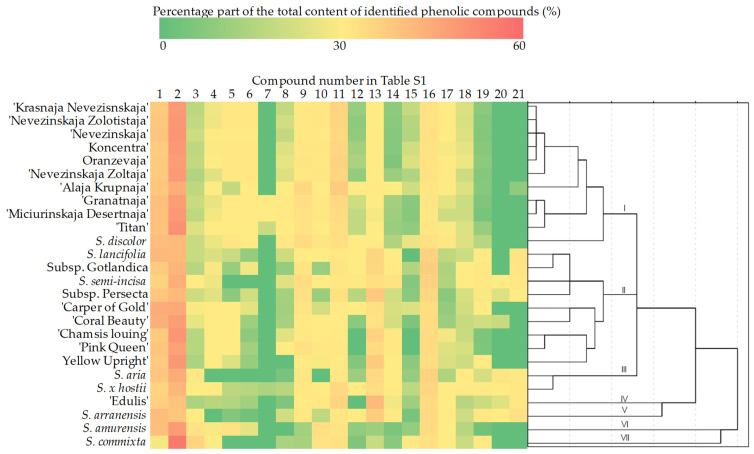
Dendrogram of hierarchical cluster analysis of *Sorbus* inflorescence samples according to the phytochemical composition and heatmap of the percentage compositions of identified phenolic compounds: 1—neochlorogenic acid; 2—chlorogenic acid; 3—cryptochlorogenic acid; 4—caffeoylshikimic acid; 5—quercetin dihexoside 1; 6—quercetin dihexoside 2; 7—quercetin pentose hexoside; 8—quercetin dihexoside 3; 9—rutin; 10—hyperoside; 11—isoquercitrin; 12—kaempferol coumaroyl glucoside; 13—quercetin 3*-O-*malonylglucoside; 14—isorhamnetin rutinoside; 15—astragalin; 16—dicaffeoylquinic acid derivative 1; 17—sexangularetin derivative; 18—dicaffeoylquinic acid derivative 2; 19—kaempferol acetyl hexoside; 20—dicaffeoylquinic acid derivative 3; 21—isorhamnetin acetyl hexoside.

Hierarchical cluster analysis was performed for the samples of *Sorbus* inflorescences, using the concentrations of identified biologically active compounds as clustering variables. The investigated *Sorbus* inflorescence samples were grouped into seven significant clusters (Figure 1).

Inflorescence samples of the ‘Krasnaja Nevezinskaja’, ‘Neveziskaja Zolotistaja’, ‘Nevezinskaja’, ‘Koncentra’, ‘Oranzevaja’, ‘Nevezinskaja Zoltaja’, ‘Alaja Krupnaja’, ‘Granatnaja’, ‘Miciurinskaja Desertnaja’, and ‘Titan’ cultivars and *S. discolor* formed the first cluster. This cluster was characterized by the highest concentration of quercetin dihexoside 2 and a high concentration of isoquercitrin, while dicaffeoylquinic acid derivative 3 was not detected and low concentrations of quercetin 3*-O-*malonylglucoside and isorhamnetin rutinoside were detected. The second cluster grouped inflorescence samples of ‘Chamsis Louing’, ‘Pink Queen’, ‘Coral Beauty’, ‘Yellow Upright’, ‘Carpet of Gold’, *S. lancifolia*, *S. hybrida* subsp. *gotlandica*, *S. semi-incisa*, and *S. hybrida* subsp. *persecta*. The samples forming the second cluster were characterized by high concentrations of quercetin 3*-O-*malonylglucoside, isorhamnetin rutinoside, caffeoylshikimic acid, dicaffeoylquinic acid derivative 1, and dicaffeoylquinic acid derivative 2 and low concentrations of quercetin dihexoside 1, quercetin dihexoside 2, and isoquercitrin. The inflorescences of *S. aria* and *S. x hostii* formed the third cluster. The corresponding inflorescence samples differed from the others in their high concentrations of dicaffeoylquinic acid derivatives and low concentrations of the other identified phenolic compounds. The inflorescences of ‘Edulis’, *S. arranensis, S. amurensis*, and *S. commixta* formed the fourth, fifth, sixth, and seventh clusters, respectively. Inflorescence samples of the ‘Edulis’ cultivar differed from the others in having highest concentrations of isoquercitrin and quercetin 3*-O-*malonylglucoside, while inflorescence samples of *S. arranensis* were characterized by the highest concentrations of kaempferol coumaroyl hexoside, isorhamnetin rutinoside, kaempferol acetylhexoside, and isorhamnetin acetylhexoside and the lowest concentrations of quercetin dihexoside 1 and quercetin dihexoside 2 (Figure 1). Inflorescence samples of *S. amurensis* differed from the others in having the highest concentrations of quercetin dihexoside 1 and hyperoside and the lowest concentration of quercetin 3*-O-*malonylglucoside. Inflorescence samples of *S. commixta* were also found to differ from the others as they showed the highest concentrations of chlorogenic acid, cryptochlorogenic acid, and astragalin and the lowest concentrations of neochlorogenic acid and rutin

### 2.3. Principal Component Analysis (PCA) of Phenolic Compounds in Sorbus L. Species and Cultivars

**Figure 2:**
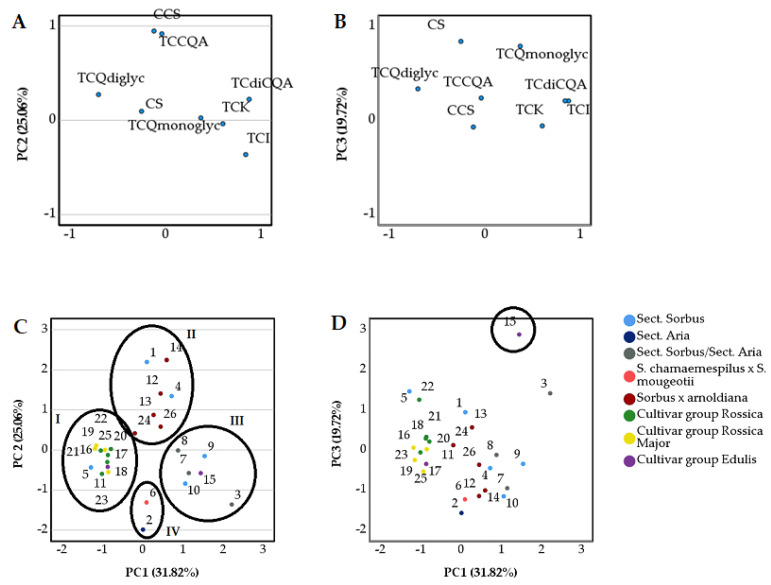
PCA loading plots (**A**,**B**) and score plots (**C**,**D**) for different *Sorbus* inflorescence samples. 1—*S. amurensis*; 2—*S. aria*; 3—*S. arranensis*; 4—*S. commixta*; 5—*S. discolor*; 6—*S. x hostii*; 7—*S. hybrida* subsp. Gotlandica; 8—*S. hybrida* subsp. Persecta; 9—*S. lancifolia*; 10—*S. semi-incisa*; 11—‘Alaja Krupnaja’; 12—‘Carpet of Gold’; 13—‘Chamsis Louing’; 14—‘Coral Beauty’; 15 –’Edulis’; 16—‘Granatnaja’; 17—‘Koncentra’; 18—‘Krasnaja Nevezisnskaja’; 19—‘Miciurinskaja Desertnaja’; 20—‘Nevezinskaja’; 21—‘Nevezinskaja Zolotistaja’; 22—‘Nevezinskaja Zoltaja’; 23—‘Oranzevaja’; 24—‘Pink Queen’; 25—‘Titan’; 26—‘Yellow Upright’.

A principal component analysis (PCA) was performed to detect similarities and differences between the analyzed samples in terms of the total concentration of caffeoylquinic acid derivatives (TCCQA), the total concentration of dicaffeoylquinic acid derivatives (TCdiCQA), the concentration of caffeoylshikimic acid (CCSA), the total concentration of quercetin monoglycosides (TCQmonoglyc), the total concentration of quercetin diglycosides (TCQdiglyc), the total concentration of kaempferol derivatives (TCK), the total concentration of isorhamnetin glycosides (TCI), and the concentration of sexangularetin derivative (CS). The PCA results based on the correlation matrix with the first principal component (PC1), the second principal component (PC2), and the third principal component (PC3) are shown in Figure 2. Three principal components explaining 76.60% of the total data variance in the datasets for the *Sorbus* inflorescences were used for the in-depth analysis. PC1 described 31.82% of the total variance in the data and correlated with positive loadings for the TCdiCQA (0.877), TCI (0.842), and TCK (0.602) and negative loadings for the TCQdiglyc (−0.699). PC2 accounted for 25.06% of the total variance and was characterized by positive loadings for the CCSA (0.947) and the TCCQA (0.917). PC3 described 19.72% of the total variance in the data and had strong positive correlations with TCQmonoglyc (0.837) and CS (0.786).

The inflorescences of *Sorbus* were clustered into four groups. Inflorescence samples of group I were closely clustered along the negative side of PC1 due to the high total concentration of quercetin diglycosides and low total concentrations of dicaffeoylquinic acids, isorhamnetin derivatives, and kaempferol derivatives. Inflorescences of group III were located on the positive side of PC1. The total concentrations of dicaffeoylquinic acids, isorhamnetin derivatives, and kaempferol derivatives that scored highly in PC1 ranged from the mean values to the highest in the corresponding samples. In contrast, the total concentration of quercetin diglycosides, which had a negative loading in PC1, was determined to be low in these inflorescence samples. Inflorescence samples of group I and group III were situated near the zero point of PC2. The concentration of caffeoylshikimic acid and total concentration of caffeoylquinic acids determined in these samples were close to the mean values. Group II was distinguished by a high concentration of caffeoylshikimic acid and a total concentration of caffeoylquinic acids that scored highly in PC2. Inflorescence samples of group IV differed from the others in their low concentration of caffeoylshikimic acid and total concentration of caffeoylquinic acids, which had positive loadings in PC2. Inflorescence samples of ‘Edulis’ were distanced from all the others and were grouped on the positive side of the PC3. This could be explained by the high concentration of sexangularetin derivative and total concentration of quercetin monoglycosides. 

## 3. Discussion

Phenolic-rich plant materials are considered as sources for added-value ingredients that could provide positive health and nutritional effects in the frame of chronic degenerative diseases resulting from the features of modern life [33]. Plants synthesize phenolic compounds as specialized metabolites against exogenous and endogenous stress factors. Furthermore, various studies have confirmed their remarkable health effects and pharmacological activities; namely, antioxidant, anti-inflammatory, cardioprotective, neuroprotective, gut microbiota-promoting, and antidiabetic effects [24,28,33,34,35]. The latter effect is one of the main medicinal effects documented for the raw materials of *Sorbus* species, with the phenolic compounds being suggested as one of the bioactive fractions [19,36,37]. The majority of ethnopharmacological, phytochemical, and nutritional studies have focused on the leaves and fruits of various *Sorbus* species and cultivars. This study elucidated the phenolic profiles of inflorescences of *Sorbus* species and cultivars. High quantitative heterogeneity across various *Sorbus* genotypes was found in the profiling. However, the prevailing compounds that were common to all *Sorbus* materials tested were present in a constant manner, with the chlorogenic and neochlorogenic acids being the key components and rutin, isoquercetin, and quercetin malonyl glucoside the prevailing flavonoids. The results are consistent with our previous studies [20,38,39,40,41] and with the studies by Olszevska et al., which, to the best of our knowledge, are to date the only phytochemical studies on the inflorescences of *Sorbus* species [23,24,27,30]. Epidemiological and clinical studies show that hydroxycinnamic acid intake is associated with reduced risk of metabolic syndrome, diabetes, and colorectal cancer. Furthermore, chlorogenic acid can modulate colonic microbiota and, via the gut–brain axis, express health benefits [42]. Hydroxycinnamic acids have anti-inflammatory [43], neuroprotective [44], antidiabetic [45], antimicrobial [46], and ultraviolet-protective [47] effects. The amount of neochlorogenic acid we observed in the inflorescence samples was close to the amount identified by Olszewska et al. (0.19–1.98%) [24,30]. Olszewska et al. found 1.78–4.17% chlorogenic acid in their inflorescence samples, and the inflorescences of *S. commixta* contained significantly higher amounts of chlorogenic acid compared to the leaves (3.92% and 0.79%, respectively) [24,30]. The species and cultivars we analyzed accumulated less chlorogenic acid (0.76–2.25%). 

*S. commixta* is a valuable species with an extraordinarily rich phenolic composition [29]. In our previous research [38,39,40], the fruits and leaves of this species were found to be exclusively separate from other species in the hierarchical cluster and PCA analyses, and they had the highest antioxidant activities. Furthermore, in this study, the inflorescences of *S. commixta* contained the greatest amounts of chlorogenic acid and, surprisingly, the lowest amounts of neochlogenic acid compared to other *Sorbus* species. However, the amounts of chlorogenic acid were sevenfold and twofold lower compared to the leaves and fruits, respectively [38,39]. The quantitative composition of phenolic acids in sweet rowanberries of the ‘Titan’, ‘Granatnaja’, ‘Alaja Krupnaja’ cultivars has been previously examined [16]. The greatest amounts of neochlorogenic and chlorogenic acids were in the fruits of ‘Granatnaja’, while the lowest amounts were determined in the fruits of ‘Alaja Krupnaja’. Our study determined corresponding patterns in the inflorescence samples of ‘Titan’, ‘Granatnaja’, and ‘Alaja Krupnaja’. Kylli et al. (2010) found the greatest amounts of neochlorogenic acid and chlorogenic acid in fruit samples from the cultivars ‘Titan’ and ‘Granatnaja’ [48]. We compared the amounts of hydroxycinnamic acids in leaves, fruits, and inflorescences (certain species overlapped in our previous studies), and the results demonstrated the superiority of the leaves and fruits. In contrast, Olszewska et al. (2010) determined that *S. commixta* inflorescences contained significantly greater amounts of total phenolics and about threefold greater amounts of chlorogenic acid compared to leaves [30]. Olszewska and Michel (2009) determined that the antioxidant activities of *S. aucuparia*, *S. aria*, and *S. intermedia* inflorescences were higher compared to the leaves and fruits. In fact, the phenolic content in the inflorescences and leaves of various *Sorbus* genotypes are comparable, and superiority strongly depends on geographic, climatic, and edaphic conditions. Further studies could target phytogeographic, phonologic, and ontogenetic profiling. The total phenolic contents in inflorescences and leaves were comparable; however, the profiles of individual flavonoids were highly variable between plant materials [24]. Flavonols are the predominant flavonoids in *Sorbus* materials [19]. Flavonols demonstrate cardioprotective [49], neuroprotective [50], hepatoprotective [51], anticancer [52], antimicrobial [53], anti-inflammatory [54], and anti-platelet [55] activities. On average, the total amount of identified flavonoids in the tested *Sorbus* inflorescences was 1.7 mg/g. The individual patterns showed high quantitative heterogeneity. Overall, the amounts of individual flavonoids in inflorescences were lower than in the leaves of the respective species (*S. arranensis*, *S. commixta*, *S. discolor*, *S. semi-incisa*) [38,40]. Olszewska determined that the total flavonoid content was superior in the inflorescences of *S. aucuparia* and *S. intermedia* compared to the leaves, and only *S. aria* leaves were superior to the inflorescences. In contrast, our results show that the total identified flavonoids in *S. aria* inflorescences were lower compared to the leaves. Hukkanen et al. (2006), for fruit samples of the ‘Titan’, ‘Granatnaja’, and ‘Alaja Krupnaja’ cultivars, determined that the greatest amount of flavonols was found in the fruits of ‘Granatnaja’, while the lowest amount was found in the fruits of ‘Alaja Krupnaja’ [16]. The pattern corresponds with the results of our present study, confirming the phytochemical impact of genotype. Quercetin derivatives have been determined to be the predominant substances in identified flavonoid complexes. The amounts of other flavonoids are significantly lower. These results are consistent with data supplied by other researchers [23]. Upon comparison of the amounts of isorhamnetin derivatives in inflorescence samples from various species and cultivars of *Sorbus*, we determined that the inflorescence samples of the subgenus *Aria* contained greater amounts of these flavonoids than the inflorescence samples of the subgenus *Sorbus*. The same patterns were detected by Polish scientists [23,29]. The amounts of isorhamnetin determined in the inflorescence samples of *S. aria* during previous research were 31–32% [56] and 44% [23] of the total flavonoid amount. Isorhamnetin could be used as an analytical marker for differentiation of raw plant materials of different subgenera. Sexangularetin is an important analytical marker of *Sorbus* inflorescence materials. In contrast to leaves, the inflorescences are abundant in sexangularetin derivatives; namely, methoxylated flavonoids [27]. Methoxylated flavonoids could be of particular scientific interest, as they have high metabolic stability and bioavailability [57]. The pattern for methoxylated flavonoids highlights the uniqueness of inflorescences compared to leaves and fruits. Our results showed that *S. discolor* had the greatest amount of sexangularetin derivatives. The genotype determined the pattern of the phenolic compounds, and the chemometric analysis distinguished two principal groups of *Sorbus* inflorescences; namely, the *Rossica* cultivar group (Table 4) and the group constituted by *S. x arnoldiana* cultivars and sect. *Sorbus* species. *S. amurensis*, *S. aria*, *S. arranensis*, *S. commixta*, and *S. x hostii* formed different, distinct groups or separate clusters, confirming their unique and particular phenolic profiles. The cultivars ‘Chamsis Louing’, ‘Coral Beauty’, and ‘Edulis’ and the species *S. amurensis*, *S. arranensis*, and *S. commixta*, remarkably, had the highest total phenolic compounds determined, ranging from 5.9 to 7.9 mg/g. 

Plant inflorescences, rich in hydroxycinnamic acids and quercetin derivatives, are potent antioxidants, possess anti-inflammatory activities, and, traditionally, are used as diuretic, diaphoretic, and anti-inflammatory agents [58,59,60,61]. Furthermore, food plants provide various raw materials that contain phytochemical compounds with health benefits. The processing of the raw materials can, depending on the final concentration of compounds, create an interface between food, medicine, and cosmetics [62]. Plant material infusions targeted as daily food intake can be employed as preventive nutrition in restoring and sustaining health and wellness [60]. *Sorbus* inflorescences are attractive plant matrixes with notable phenolic profiles. Modulation of the stages of genotype selection, extraction, and processing can unlock their potential multi-industrial applications in future preparations.

## 4. Materials and Methods

### 4.1. Plant Material

In May 2012, inflorescence samples from *Sorbus* species and cultivars were collected in the southeastern region of Lithuania at the Botanical Garden of Vilnius University (54°43′48″ N 25°23′56″ E) for a phytochemical analysis. Eight rowan species (*S. amurensis*, *S. aria*, *S. arranensis* Hedl. (voucher specimen no. 3641), *S. commixta* (voucher specimen no. 3652), *S. discolor* Maxim. (voucher specimen no. 3653), *S. lancifolia* Hedl. (voucher specimen no. 3661), *S. semi-incisa* (Borbas) Borbas (voucher specimen no. 3663), and *S. x hostii* (J.Jacq.) K.Koch (voucher specimen no. 3659)), sixteen cultivars (‘Alaja Krupnaja’ (voucher specimen no. 3423), ‘Nevezinskaja’ (voucher specimen no. 3428), ‘Oranzevskaja’ (voucher specimen no. 3425), ‘Titan’ (voucher specimen no. 3424), ‘Granatnaja’ (voucher specimen no. 3433), ‘Carpet of Gold’ (voucher specimen no. 2895), ‘Chamsis Louing’ (voucher specimen no. 2887), ‘Coral Beauty’ (voucher specimen no. 2896), ‘Edulis’ (voucher specimen no. 3645), ‘Koncentra’ (voucher specimen no. 3435), ‘Krasnaja Nevezinskaja’ (voucher specimen no. 3648), ‘Miciurinskaja Desertnaja’ (voucher specimen no. 2897), ‘Nevezinskaja Zolotistaja’ (voucher specimen no. 3653), ‘Neveziskaja Zoltaja’ (voucher specimen no. 3442), ‘Pink Queen’ (voucher specimen no. 2888), ‘Yellow Upright’ (voucher specimen no. 2889)), and two *S. hybrida* subspecies (subsp. Gotlandica (voucher specimen no. 3656), subsp. Persecta (voucher specimen no. 3657)) were collected.

The collected raw plant material samples were dried at room temperature and stored in a dark, dry place. The research results were re-calculated for absolutely dry raw plant material.

### 4.2. Materials and Reagents

Analytical and chromatographic grade reagents were used for this study: gradient-grade acetonitrile, MS-grade formic acid, neochlorogenic acid, cryptochlorogenic acid, quercetin 3*-O-*(6″*-O-*malonyl)-β-D-glucoside (quercetin 3*-O-*malonylglucoside hereinafter), isorhamnetin 3*-O-*rutinoside (Sigma-Aldrich GmbH, Steinheim, Germany), HPLC-grade 99.8% trifluoracetic acid, chlorogenic acid, HPLC-grade hyperoside, HPLC-grade isoquercitrin, HPLC-grade rutin, astragalin (Carl Roth GmbH, Karlsruhe, Germany), and 96.3% ethanol (Stumbras SC, Kaunas, Lithuania). The purified deionized water (18.2 mΩ/cm) was produced using a Millipore (Burlington, MA.,USA) water purification system.

### 4.3. Sample Preparation

For qualitative and quantitative analysis of flavonoid glycosides and phenolic acids, the rowan inflorescence samples were crushed into particles and passed through a 355 μm sieve. The samples weighed around 0.25 g (accurate sample). The weighed raw plant material sample was then placed into a conical flask with 25 mL of 50% ethanol. The extraction was performed by submerging the materials in a BioSonic UC100 ultrasonic bath (Cuyahoga Falls, Ohio, USA) for 20 min. The obtained extract was filtered through a paper filter into a 25 mL volumetric flask and adjusted accordingly to a volume with 50% ethanol. Before high-performance liquid chromatography (HPLC) analysis, extracts were filtered through a membrane filter with a pore size of 0.22 μm (Carl Roth GmbH, Karlsruhe, Germany).

### 4.4. HPLC Analysis

The analysis of extracts was performed using a previously optimized and validated (Gaivelyte et al., 2014) HPLC method. Briefly, quantitative analysis was performed using a Waters 2695 Alliance system (Waters, Milford, MA, USA) with a Waters 996 photodiode array detector. Separation was performed using an ACE (ACT, Aberdeen, UK) column (C18, 150 mm × 4.6 mm, particle size: 3 μm). The mobile phase of the optimized chromatographic method consisted of eluents A (0.05% trifluoracetic acid) and B (acetonitrile). The gradient variation was as follows: 0–5 min—12% B, 5–50 min—12–30% B, 50–51 min—30–90% B, 51–56 min—90% B, 57 min—12% B. The eluent flow rate was 0.5 mL/min, and the injection volume was 10 μL. The column was temperature-controlled, and the temperature was maintained at 25 °C. Calibration curves of compounds identified in the rowan inflorescence extracts were compiled; these curves were used for quantitative assessment. Quantification of caffeoylshikimic acid and dicaffeoylquinic acid derivatives was performed using a chlorogenic acid calibration curve, quantification of quercetin diglycosides using a rutin calibration curve, quantification of kaempferol derivatives using an astragalin calibration curve, and quantification of isoramnetin and sexangularetin derivatives using an isorhamnetin 3*-O-*rutinoside calibration curve. Concentrations of phenolic acids were calculated at a wavelength of 325 nm, while the concentrations of flavonoids were calculated at a wavelength of 350 nm.

### 4.5. UPLC-ESI-MS Conditions

Separation of phenolic compounds was carried out with an Acquity H-class UPLC system (Waters, USA) equipped with a Waters 2998 photodiode array detector, and a triple quadrupole tandem mass spectrometer (Xevo, Waters, USA) with an electrospay ionisation source (ESI) was used to obtain MS/MS data. A YMC Triart C18 (100 × 2.0 mm 1.9 µm) column was used for analysis. The column temperature was maintained at 40 °C. Gradient elution was performed with a mobile phase consisting of 0.1% formic acid water solution (solvent A) and acetonitrile (solvent B), with the flow rate set to 0.5 mL/min. The linear gradient variation was as follows: 0–1 min—5% B, 1–5 min—5–30% B, 5–7 min—30–50% B, 7–7.5 min—50–100% B, 7.5–8 min—100%, 8–8.1 min—100–5% B, 8.1–10 min—5% B. Negative electrospray ionization was applied for analysis with the following settings: capillary voltage—2 kV, source temperature—150 °C, desolvation temperature—400 °C, desolvation gas flow—700 L/h, cone gas flow—20 L/h. Collision energy and cone voltage were optimized for each compound separately.

### 4.6. Statistical Analysis

Phenolic compound content was expressed as the mean ± standard deviation (SD) of three replicates. The statistical data analysis was evaluated by applying ANOVA with a Tukey HSD post hoc test. Statistically significantly different means were marked with different letters. Differences were considered statistically significant when *p* < 0.05. In accordance with the quantitative composition of the identified compounds, the tested samples were compared using the method of hierarchical cluster analysis with squared Euclidean distances. Principal component analysis was performed taking into account factors with eigenvalues higher than 1. The data were processed using Microsoft Office Excel 2010 (Microsoft, JAV) and SPSS 20 software. 

## 5. Conclusions

The current study provides a comprehensive analysis of the phenolic profiles of 27 *Sorbus* genotypes and complements knowledge regarding the patterns of specialized metabolites in *Sorbus* plant materials. The findings showed that the inflorescence extracts were abundant in hydroxycinnamic acids and flavonol derivatives. The leaves and inflorescences were comparable in phenolic contents, and the significant superiority of inflorescences was not observed. Phenolic fingerprint profiles and sexangularetin derivatives could serve as markers in authenticity studies and quality control schemes. The species *S. amurensis*, *S. arranensis*, *S. commixta*, and *S. discolor* and cultivars ‘Chamsis Louing’, ‘Coral Beauty’, and ‘Edulis’ could be target genotypes for production of smart and innovative inflorescence matrix-based ingredients.

## Figures and Tables

**Table 1:** Concentrations of phenolic acids (µg/g DW) in *Sorbus* inflorescences.

Species/Cultivars	Compound						
	Neochlorogenic Acid	Chlorogenic Acid	Cryptochlorogenic Acid	Caffeoylshikimic Acid	Dicaffeoylquinic Acid Derivative 1	Dicaffeoylquinic Acid Derivative 2	Dicaffeoylquinic Acid Derivative 3
*S. amurensis*	1701 ± 44 ab ^1^	2973 ± 147 ab	463 ± 17 b	131 ± 2 b	721 ± 42 a	72 ± 1 a	nd ^2^
*S. aria*	432 ± 96 ijk	691 ± 126 kl	81 ± 10 cd	nd	285 ± 39 jkl	58 ± 0 g	113 ± 16 a
*S. arranensis*	1397 ± 28 bc	1161 ± 22 ijkl	120 ± 2 c	nd	606 ± 0 abcd	73 ± 1 a	120 ± 4 a
*S. commixta*	95 ± 12 k	3154 ± 250 a	721 ± 86 a	126 ± 7 bc	401 ± 40 fghijk	67 ± 2 abcdef	nd
*S. discolor*	1118 ± 15 cde	1167 ± 60 ijkl	55 ± 0 cd	74 ± 1 fg	312 ± 1 ijkl	67 ± 0 abcdef	nd
*S. x hostii*	307 ± 1 jk	632 ± 32l	47 ± 1 d	57 ± 0 gh	298 ± 18 ijkl	60 ± 1 fg	65 ± 1 c
*S. hybrida* subsp. Gotlandica	661 ± 43 ghij	1060 ± 70 jkl	37 ± 1 d	65 ± 2 fgh	612 ± 65 abc	69 ± 3 abcd	nd
*S. hybrida* subsp. Persecta	1040 ± 4 cdef	1305 ± 42 ghijkl	63 ± 3 cd	76 ± 1 f	598 ± 12 abcde	69 ± 1 abcde	73 ± 3 c
*S. lancifolia*	1313 ± 41 cd	1260 ± 143 hijkl	59 ± 1 cd	68 ± 2 fgh	679 ± 95 a	69 ± 2 abc	nd
*S. semi-incisa*	464 ± 12 hij	1068 ± 41 jkl	66 ± 5 cd	56 ± 0 h	487 ± 9 bcdefgh	73 ± 0 a	69 ± 0c
‘Alaja Krupnaja’	819 ± 6 efgh	1340 ± 68 ghijkl	45 ± 4 d	77 ± 1 f	187 ± 15 l	60 ± 1 fg	nd
‘Carpet of Gold’	1774 ± 244 a	1793 ± 293 defghij	96 ± 15	127 ± 9 bc	467 ± 48 cdefghi	70 ± 1 abc	nd
‘Chamsis Louing’	1042 ± 67 cedf	2587 ± 156 abc	59 ± 0 cd	111 ± 5 cd	590 ± 42 abcde	71 ± 2 ab	nd
‘Coral Beauty’	1871 ± 174 a	2406 ± 144 cd	95 ± 0 cd	157 ± 6 a	567 ± 49 abcdef	71 ± 2 ab	72 ± 1
‘Edulis’	979 ± 139 defg	1407 ± 207 fghijk	56 ± 8 cd	66 ± 4 fgh	651 ± 88 ab	65 ± 7 abcdefg	96 ± 4b
‘Granatnaja’	1134 ± 30 cde	1959 ± 34 cdefgh	59 ± 0 cd	78 ± 0 f	212 ± 2 l	61 ± 0 fg	nd
‘Koncentra’	688 ± 23 fghi	1663 ± 41 efghij	42 ± 0 d	71 ± 1 fgh	247 ± 3 kl	61 ± 1 defg	nd
‘Krasnaja Nevezisnskaja’	808 ± 104 efgh	1838 ± 266 defhgi	46 ± 4 d	74 ± 4 fg	264 ± 42 jkl	63 ± 1 cdefg	nd
‘Miciurinskaja Desertnaja’	1094 ± 8 cde	2038 ± 103 cdefg	52 ± 1 cd	82 ± 0 ef	192 ± 8 l	60 ± 1 fg	nd
‘Nevezinskaja’	921 ± 63 efg	1994 ± 169 cdefgh	56 ± 4 cd	82 ± 2 ef	331 ± 34 ghijkl	66 ± 2 abcdef	nd
‘Nevezinskaja Zolotistaja’	899 ± 132 efg	1973 ± 218 cdefgh	51 ± 2 cd	76 ± 4 f	296 ± 24 ijkl	64 ± 0 bcdefg	nd
‘Nevezinskaja Zoltaja’	955 ± 25 efg	2195 ± 50 cde	49 ± 1 cd	77 ± 0 f	317 ± 3 hijkl	66 ± 1 abcdef	nd
‘Oranzevaja’	661 ± 44 ghij	1496 ± 147 efghij	42 ± 3 d	70 ± 2 fgh	226 ± 7 l	62 ± 1 defg	nd
‘Pink Queen’	1040 ± 44 cdef	1896 ± 212 cdefghi	50 ± 4 cd	104 ± 1 d	498 ± 36 bcdefg	70 ± 2 abc	nd
‘Titan’	950 ± 112 efg	1957 ± 232 cdefgh	61 ± 10 cd	82 ± 3 ef	222 ± 12l	61 ± 0 efg	nd
‘Yellow Upright’	889 ± 99 efg	2094 ± 358 cdef	43 ± 4 d	112 ± 11 cd	433 ± 65 defghij	62 ± 1 defg	nd

^1^ Averages marked in different letters in the columns show statistically significant difference (at *p* < 0.05); ^2^ nd—not detected.

**Table 2:** Concentrations of quercetin derivatives (µg/g DW) in *Sorbus* inflorescences.

Species/Cultivars	Compounds							
	Quercetin Dihexoside 1	Quercetin Dihexoside ^2^	Quercetin Pentose Hexoside	Quercetin Dihexoside 3	Rutin	Hyperoside	Isoquercitrin	Quercetin 3*-O-*Malonylglucoside
*S. amurensis*	437 ± 12 a ^1^	112 ± 6 fg	nd	nd	54 ± 2 p	567 ± 11 a	399 ± 15 fg	50 ± 8 i
*S. aria*	nd^2^	nd	nd	6 ± 0 j	105 ± 7 no	nd	88 ± 6 l	223 ± 18 fg
*S. arranensis*	23 ± 1 kl	11 ± 0 pr	nd	75 ± 1 de	242 ± 2 gh	64 ± 1 lmn	25 ndhi	994 ± 5 b
*S. commixta*	nd	nd	nd	46 ± 1 fgh	44 ± 1 p	336 ± 21 b	389 ± 23 g	65 ± 1 hi
*S. discolor*	261 ± 8 b	124 ± 5 ef	nd	154 ± 8 a	439 ± 19 a	261 ± 5 c	356 ± 2 g	108 ± 1 fghi
*S. x hostii*	25 ± 1 kl	24 ± 0 op	20 ± 1c	23 ± 0 i	94 ± 3 op	63 ± 1 lmn	256 ± 6 hi	210 ± 3 fgh
Subsp. Gotlandica	23 ± 1 kl	63 ± 2 k	nd	23 ± 0 i	204 ± 2 hij	24 ± 0 no	205 ± 1 ijk	442 ± 3 d
Subsp. Persecta	25 ± 1 kl	65 ± 1 jk	nd	39 ± 2 h	379 ± 8 b	33 ± 1 mno	349 ± 3 g	970 ± 12 b
*S. lancifolia*	59 ± 3 j	29 ± 2 no	nd	62 ± 4 ef	126 ± 12 mno	125 ± 9 ghij	199 ± 21 ijk	604 ± 70 c
*S. semi-incisa*	nd	nd	nd	50 ± 1 fgh	290 ± 10	66 ± 3 lm	122 ± 3 kl	262 ± 17 ef
‘Alaja Krupnaja’	47± 2 jk	101 ± 1 gh	nd	78 ± 1 d	465 ± 2 a	97 ± 2 ijkl	713 ± 12 b	133 ± 0 fghi
‘Carpet of Gold’	113 ± 10 g	82 ± 4 ij	nd	42 ± 1 gh	292 ± 16 def	72 ± 5 klm	173 ± 10 ijkl	385 ± 14 de
‘Chamsis Louing’	111 ± 3 gh	30 ± 1 no	nd	88 ± 0 cd	213 ± 1 hij	151 ± 0 efg	179 ± 4 ijkl	851 ± 35 b
‘Coral Beauty’	124 ± 1 fg	42 ± 1 lmn	nd	49 ± 1 fgh	176 ± 0 ijkl	124 ± 1 ghij	159 ± 0 jkl	400 ± 1 de
‘Edulis’	61 ± 4 j	49 ± 2 klm	nd	22 ± 0 i	164 ± 11 jklm	257 ± 21 c	874 ± 48 a	1670 ± 115 a
‘Granatnaja’	118 ± 1 g	126 ± 3 ef	90 ± 3 a	153 ± 5 a	357 ± 9 b	182 ± 8 def	526 ± 15 de	94 ± 8 ghi
‘Koncentra’	166 ± 5 de	160 ± 4 bc	nd	58 ± 1 fg	185 ± 6 ijkl	172 ± 2 def	512 ± 2 de	97 ± 0 ghi
‘Krasnaja Nevezisnskaja’	181 ± 17 d	170 ± 15 b	nd	50 ± 7 fgh	152 ± 15 klmn	207 ± 17 d	593 ± 60 cd	113 ± 8 fghi
‘Miciurinskaja Desertnaja’	123 ± 1 fg	120 ± 1 ef	90 ± 1 a	141 ± 3 a	306 ± 5 cde	182 ± 6 de	480 ± 9 ef	85 ± 3 ghi
‘Nevezinskaja’	131 ± 3 fg	138 ± 2 de	nd	51 ± 0 fgh	192 ± 10 ijk	129 ± 2 ghi	494 ± 11 e	96 ± 7 ghi
‘Nevezinskaja Zolotistaja’	168 ± 11 de	152 ± 12 bcd	nd	60 ± 7 ef	173 ± 18 ijklm	200 ± 19 d	559 ± 51 de	94 ± 11 ghi
‘Nevezinskaja Zoltaja’	216 ± 2 c	202 ± 3 a	nd	75 ± 5 de	222 ± 17 hi	254 ± 12 c	654 ± 33 bc	130 ± 1 fghi
‘Oranzevaja’	144 ± 5 ef	150 ± 1 cd	nd	59 ± 2 f	206 ± 12 hij	141 ± 7 fgh	520 ± 21 de	82 ± 0 ghi
‘Pink Queen’	120 ± 7 fg	38 ± 1 lmno	nd	113 ± 10 b	341 ± 27 bcd	206 ± 19 d	204 ± 17 ijk	531 ± 66 cd
‘Titan’	87 ± 6 hi	89 ± 5 hi	51 ± 2 b	96 ± 7 c	249 ± 19 fgh	109 ± 10 hijk	328 ± 23 gh	85 ± 8 ghi
‘Yellow Upright’	70 ± 11 ij	31 ± 4 mno	nd	nd	139 ± 15 lmno	88 ± 10 jkl	151 ± 16 jkl	866 ± 111 b

^1^ Averages marked in different letters in the columns show statistically significant difference (at *p* < 0.05); ^2^ nd—not detected.

**Table 3:** Concentrations of derivatives of the other flavonoids (µg/g DW) in *Sorbus* inflorescences.

Species/Cultivars	Compounds					
	Kaempferol Coumaroyl Glucoside	Isorhamnetin Rutinoside	Astragalin	Sexangularetin Derivative	Kaempferol Acetyl Hexoside	Isorhamnetin Acetyl Hexoside
*S. amurensis*	40 ± 9 fghi ^1^	23 ± 6 gh	44 ± 3 de	148 ± 20 bc	14 ± 1 i	nd ^2^
*S. aria*	14 ± 1 jk	48 ± 7 defg	13 ± 1 j	19 ± 2 k	28 ± 0 hi	74 ± 6 e
*S. arranensis*	141 ± 0 a	186 ± 3 a	15 ± 0 hij	135 ± 7 bcd	238 ± 7 a	421 ± 1 a
*S. commixta*	19 ± 0 jk	25 ± 1 gh	185 ± 1 a	123 ± 10 cde	159 ± 19 bc	nd
*S. discolor*	107 ± 0 c	41 ± 7 efgh	73 ± 2 c	199 ± 11 a	45 ± 2 gh	nd
*S. x hostii*	48 ± 2 efgh	59 ± 1 cdef	25 ± 2 g	44 ± 6 hijk	67 ± 5 fg	83 ± 1
Subsp. Gotlandica	75 ± 1 d	112 ± 1 b	35 ± 3 f	32 ± 2 ijk	133 ± 4 cd	183 ± 1 c
Subsp. Persecta	51 ± 5 efg	66 ± 3 cde	24 ± 3 gh	24 ± 1 jk	90 ± 7 ef	139 ± 1 d
*S. lancifolia*	117 ± 22 c	111 ± 19 b	nd	54 ± 13 ghi	125 ± 19 d	262 ± 24 b
*S. semi-incisa*	120 ± 4 bc	198 ± 3 a	14 ± 1 ij	32 ± 1 ijk	133 ± 1 cd	123 ± 0 d
‘Alaja Krupnaja’	76 ± 1 d	76 ± 3 cd	53 ± 0 d	76 ± 4 fg	17 ± 1 i	22 ± 1 f
‘Carpet of Gold’	101 ± 3 c	74 ± 13 cd	77 ± 4 bc	32 ± 0 ijk	111 ± 1 de	nd
‘Chamsis Louing’	nd	207 ± 19 a	nd	10 ndef	19 ± 0 hi	nd
‘Coral Beauty’	29 ± 2 hij	81 ± 4 bc	19 ± 0 ghij	41 ± 2 hijk	78 ± 1 f	nd
‘Edulis’	nd	109 ± 16 b	46 ± 5 de	147 ± 4 bc	79 ± 2 f	254 ± 23 b
‘Granatnaja’	63 ± 1 de	38 ± 1 efgh	23 ± 2 ghi	76 ± 3 fg	17 ± 2 i	nd
‘Koncentra’	31 ± 2 ghij	17 ± 4 gh	51 ± 1 de	69 ± 2 gh	18 ± 1 i	nd
‘Krasnaja Nevezisnskaja’	27 ± 2 hij	22 ± 1 gh	51 ± 2 de	111 ± 8 de	21 ± 1 hi	nd
‘Miciurinskaja Desertnaja’	54 ± 0 ef	35 ± 3 efgh	21 ± 1 ghij	61 ± 1 ghi	13 ± 2 i	nd
‘Nevezinskaja’	26 ± 0 ij	2 ndgh	43 ± 3 ef	125 ± 12 cde	17 ± 0 i	nd
‘Nevezinskaja Zolotistaja’	23 ± 4 ij	22 ± 1 gh	45 ± 3 de	118 ± 5 cde	19 ± 1 hi	nd
‘Nevezinskaja Zoltaja’	30 ± 1 hij	35 ± 5 efgh	48 ± 1 de	165 ± 5 a	21 ± 1 hi	nd
‘Oranzevaja’	32 ± 1 ghij	13 ± 1 h	53 ± 0 d	100 ± 5 ef	19 ± 1 hi	nd
‘Pink Queen’	nd	190 ± 14 a	nd	74 ± 9 fg	17 ± 1 i	nd
‘Titan’	47 ± 4 efgh	28 ± 3 fgh	23 ± 2 ghi	77 ± 2 fg	28 ± 2 hi	nd
‘Yellow Upright’	19 ± 1 ijk	113 ± 6 b	24 ± 3 gh	69 ± 9 gh	136 ± 16 cd	nd

^1^ Averages marked in different letters in the columns show statistically significant difference (at *p* < 0.05); ^2^ nd—not detected.

**Table 4:** *Sorbus* L. species and cultivars.

	Species and Cultivars	
1	*S. amurensis*	*Sorbus aucuparia* subsp*. pohuashanensis*
2	*S. aria*	sect. *Aria* Pers.
3	*S. arranensis*	sect. *Sorbus*/sect. *Aria*.
4	*S. commixta*	sect. *Sorbus*
5	*S. discolor*	*Sorbus* subg. *Albocarmesinae*
6	*S. x hostii*	*S. chamaemespilus* (L.Crantz. *× S. mougeotii* Soy.—Willem ex Godr.)
7	*S. hybrida* subsp. *gotlandica*	sect. *Sorbus*/sect. *Aria*. (*S. aucuparia × S. rupicola*)
8	*S. hybrida* subsp. *persecta*	sect. *Sorbus*/sect. *Aria*. (*S. aucuparia × S. rupicola*)
9	*S. lancifolia*	sect. *Sorbus*
10	*S. semi-incisa*	sect. *Sorbus*
11	‘Alaja Krupnaja’	Cultivar of the group Rossica Major. *S. aucuparia × Pyrus sp. × S. aucuparia var. moravica*
12	‘Carpet of Gold’	*Sorbus × arnoldiana* [*Sorbus aucuparia × Sorbus discolor*]
13	‘Chamsis Louing’	*Sorbus × arnoldiana* [*Sorbus aucuparia × Sorbus discolor*]
14	‘Coral Beauty’	*Sorbus × arnoldiana* [*Sorbus aucuparia × Sorbus discolor*]
15	‘Edulis’	Cultivar of the group Edulis *S. aucuparia* var. *dulcis*, *S. aucuparia* var. *edulis*
16	‘Granatnaja’	Cultivar of the group Rossica Major (*S. aucuparia × Crataegus sanguinea* Pall.)
17	‘Koncentra’	Cultivar of the group Edulis
18	‘Krasnaja Nevezinskaja’	Cultivar of the group Rossica
19	‘Miciurinskaja Desertnaja’	Cultivar of the group Rossica Major ([*S. aucuparia × Aronia melanocarpa* (Michx.) Elliott.] *× Mespilus germanica* L.)
20	‘Nevezinskaja’	Cultivar of the group Rossica
21	‘Nevezinskaja Zolotistaja’	Cultivar of the group Rossica
22	‘Nevezinskaja Zoltaja’	Cultivar of the group Rossica
23	‘Oranzevaja’	Cultivar of the group Rossica
24	‘Pink Queen’	*Sorbus × arnoldiana* [*Sorbus aucuparia × Sorbus discolor*]
25	‘Titan’	Cultivar of the group Rossica Major (*S. aucuparia × Sorbaronia alpina* (*S. aria × Aronia arbutifolia*) *×* mixture of pollen from *Malus* sp. and *Pyrus* sp.
26	‘Yellow Upright’	*Sorbus × arnoldiana* [*Sorbus aucuparia × Sorbus discolor*]

## Data Availability

All data generated during this study are included in this article.

## Supplementary Materials

The following supporting information can be downloaded at: https://www.mdpi.com/article/10.3390/plants11243421/s1, Figure S1: *Sorbus* L. inflorescence phytochemical profiles; Table S1: Identified phenolic compounds in *Sorbus* L. inflorescences.
