# Supplementary material for: Phytoprofiling of Sorbus L. Inflorescences: A Valuable and Promising Resource for Phenolics
Source: Plants (Basel). 2022 Dec 7;11(24):3421. doi: 10.3390/plants11243421 (PMC9780963; doi:10.3390/plants11243421)
Supplement: Supplementary file 1 [file plants-11-03421-s001.zip › plants-2054980-supplementary.pdf]

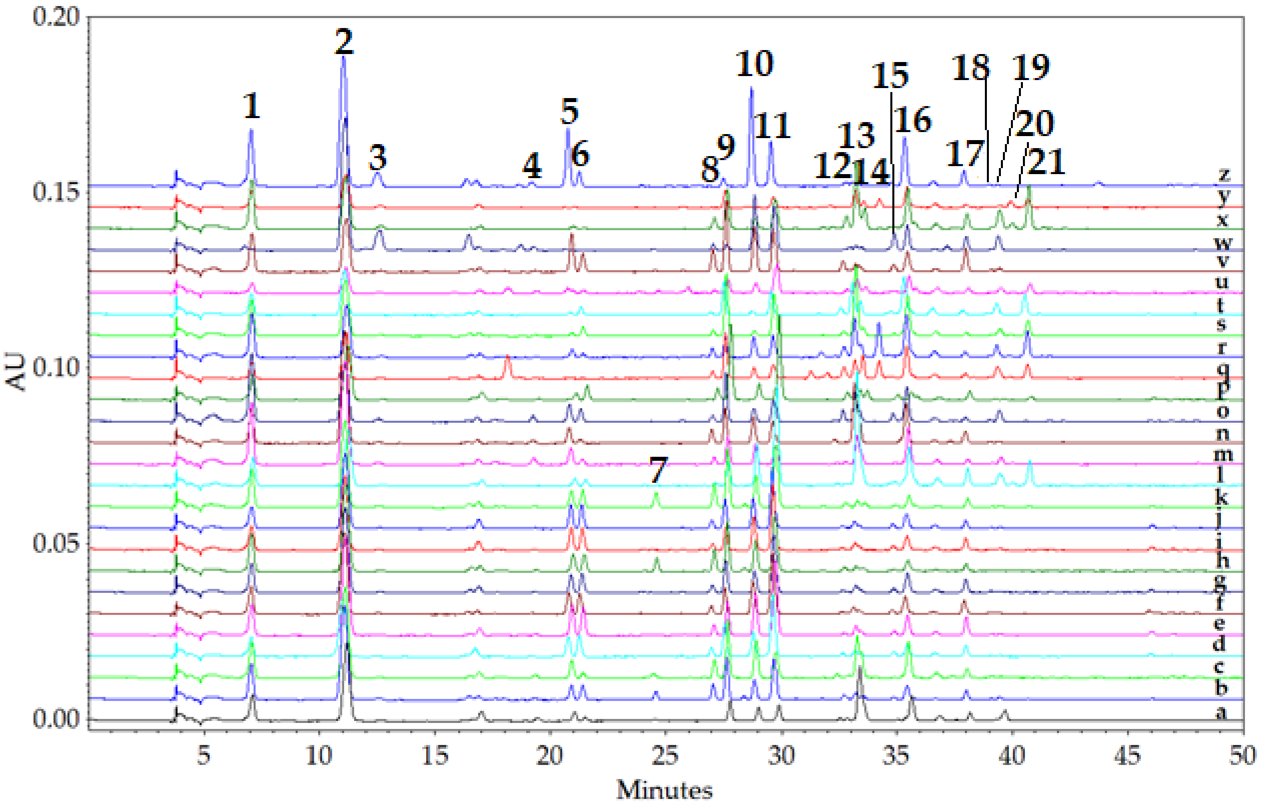

Figure S1. *Sorbus* L. inflorescences phytochemical HPLC profiles (λ=350 nm): z – *S. amurensis*; y – *S. aria*; x – *S. arranensis*; w – *S. commixta*; v – *S. discolor*;u – *S. x hostii*; t – *S. hybrida* subsp. Gotlandica; s – *S. hybrida* subsp. Persecta; r – *S. lancifolia*; q – *S. semi-incisa*; p – 'Alaja Krupnaja'; o – 'Carpet of Gold'; n – 'Chamsis louing'; m – 'Coral Beauty'; l –'Edulis'; k – 'Granatnaja'; j – 'Koncentra'; i – 'Krasnaja Nevezisnskaja'; h – 'Miciurinskaja Desertnaja'; g – 'Nevezinskaja'; f – 'Nevezinskaja Zolotistaja'; e – 'Nevezinskaja Zoltaja'; d – 'Oranzevaja'; c – 'Pink Queen'; b – 'Titan'; a – 'Yellow Upright'.

1 - Neochlorogenic acid; 2 - Chlorogenic acid; 3 - Cryptochlorogenic acid; 4 - Caffeoylshikimic acid; 5 - Quercetin dihexoside 1; 6 - Quercetin dihexoside 2; 7 - Quercetin pentose hexoside; 8 - Quercetin dihexoside 3; 9 – Rutin; 10- Hyperoside; 11 – Isoquercitrin; 12 - Kaempferol coumaroyl glucoside; 13 - Quercetin 3-O-malonylglucoside; 14 - Isorhamnetin rutinoside; 15 – Astragalin; 16 - Dicaffeoylquinic acid derivative 1; 17 - Sexangularetin derivative; 18 - Dicaffeoylquinic acid derivative 2; 19 - Kaempferol acetyl hexoside; 20 - Dicaffeoylquinic acid derivative 3; 21 - Isorhamnetin acetyl hexoside.

Table S1. Identified phenolic compounds of *Sorbus* L. inflorescences.

Peak No.	Retention time,min	Compound	λmax	UPLC-ESI-MS	
				$[M-H]^-$	Fragments
1	7.11	Neochlorogenic acid	325	353	191
2	11.20	Chlorogenic acid	325	353	191
3	12.71	Cryptochlorogenic acid	325	353	191
4	19.40	Caffeoylshikimic acid	328	335	135, 161, 179
5	20.97	Quercetin dihexoside 1	253, 354	625	301
6	21.43	Quercetin dihexoside 2	253, 354	625	301
7	24.69	Quercetin pentose hexoside	255, 354	595	301
8	27.16	Quercetin dihexoside 3	253, 354	609	301
9	27.74	Rutin	253, 354	609	301
10	28.96	Hyperoside	253, 354	463	301
11	29.79	Isoquercitrin	253, 354	463	301
12	32.83	Kaempferol coumaroyl glucoside	264, 346	593	285
13	33.27	Quercetin 3-O-malonylglucoside	253, 354	549	301, 505
14	33.63	Isorhamnetin rutinoside	253, 354	623	315, 477
15	34.93	Astragalin	264, 346	447	285
16	35.51	Dicaffeoylquinic acid derivative 1	327	515	191, 353
17	38.05	Sexangularetin derivative	270, 325, 354	519	299, 315
18	39.14	Dicaffeoylquinic acid derivative 2	326	515	191, 353
19	39.46	Kaempferol acetyl hexoside	264, 346	489	285
20	40.00	Dicaffeoylquinic acid derivative 3	327	515	191, 353
21	40.73	Isorhamnetin acetyl hexoside	253, 354	519	315
